# The complete chloroplast genome of *Pedicularis alaschanica* (Orobanchaceae)

**DOI:** 10.1080/23802359.2019.1623723

**Published:** 2019-07-15

**Authors:** Chenyu Wu, Dongming Fang, Jinpu Wei, Xuebing Wang, Xiaoli Chen

**Affiliations:** a BGI-Shenzhen, Shenzhen, China;; b China National GeneBank, BGI-Shenzhen, Shenzhen, China;; c State Key Laboratory of Agricultural Genomics, BGI-Shenzhen, Shenzhen, China

**Keywords:** Complete chloroplast genome, *Pedicularis alaschanica*, phylogenetic relationship, hemiparasites

## Abstract

The complete chloroplast genome sequence of *Pedicularis alaschanica* was determined and described. The complete chloroplast was 146,989 bp in length with typical quadripartite structure and overall GC content of 38.4%, which encompassed 68 protein-coding genes, 22 tRNAs, 4 rRNAs, and 11 pseudogenes. The functions of *ndh* genes were lost. The phylogenetic analysis indicated that *P. alaschanica* was close to other species of *Pedicularis*. This study would contribute to enrich the *Pedicularis* chloroplast genome resource and promote the biological research.

*Pedicularis* is the largest genus in Orobanchaceae, with approximately 600 species distributed in the northern hemisphere, while about 352 species (271 endemic) occur in China (Hong et al. 1998). Species of *Pedicularis* are biennial or perennial hemiparasitic herbs (Yu et al. 2015), while the evolution of chloroplast genomes of Orobanchaceae gave us a mechanistic model of evolutionary rate variation when transferred from autotrophic to nonphotosynthetic lifestyle (Wicke et al. 2013; Cusimano and Wicke 2016; Wicke et al. 2016). In this study, we reported the first complete chloroplast genome of *P. alaschanica* and the phylogenetic study.

The voucher specimen were collected from Dulan, Qinghai province, China (35° 32′ 56.03″N, 98° 02′ 17.05″E) and deposited in the Herbarium of China National Genebank (DL0048). Total genomic DNA was extracted from fresh leaves using a CTAB DNA-extraction protocol. Pair-end reads were sequenced on BGISEQ-500 sequencer at the BGI, Shenzhen. Total of 129.475 Gb raw WGS data were generated. The chloroplast genome was assembled using NOVOPlasty (v 2.7.2) (Dierckxsens et al. 2017), which was annotated using the software CpGAVAS (Liu et al. 2012). The complete chloroplast genome sequence of *P. alaschanica* has been deposited in CNSA under the accession number CNA0002395, and NCBI under the accession number MK795426.

The complete chloroplast genome sequence of *Pedicularis alaschanica* was 146,989 bp, containing two inverted-repeat regions (49,824 bp), a large single-copy region (82,736 bp), and a small single-copy region (14,429 bp), which consisted of 68 protein-coding genes, 22 tRNAs, and 4 rRNAs. All 11 of the *ndh* genes were pseudogenized (*ndhB*, *ndhD*, *ndhE*, *ndhH*, *ndhG*, *ndhJ*, and *ndhK*), truncated (*ndhC* and *ndhI*), or deleted (*ndhA*, *ndhF*, *ndhG* and *ndhI*). The average GC content of the overall genome was 38.4%, compared with 36.33% for the LSC and 43.57% for the IR region.

To further validate the assembled sequence, we presented the phylogenetic estimation with MrBayes (Ronquist and Huelsenbeck 2003) for *P. alaschanica* based on molecular data of relatively 14 related chloroplast genomes from Genbank database, using all CDS sequences, GTR model, 100,000 generation, and gamma rates across sites. Our analysis was based on up to 22,320 bp of sequence data per species from 32 genes and yielded a well-supported phylogenetic estimation for *Pedicularis* with 100% bootstrap support (Figure 1). The phylogenetic analysis showed that *P. alaschanica* was more closely related to other reported species in *Pedicularis*. The complete chloroplast genome of *P. alaschanica* would provide more valuable information to study on the evolution of genus *Pedicularis*.

**Figure 1.**
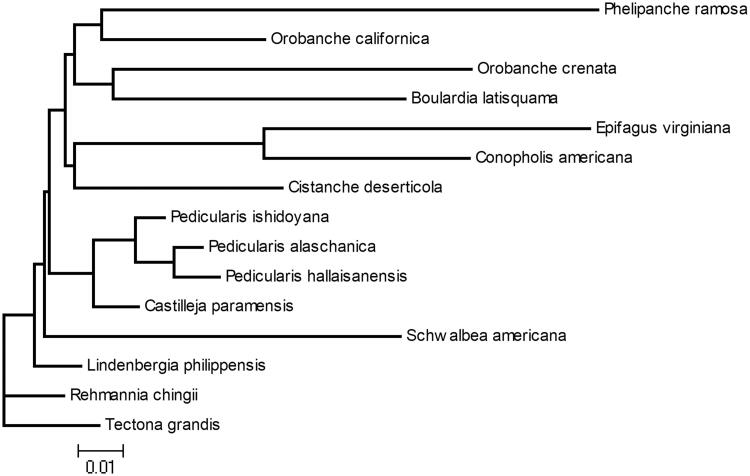
Phylogenetic tree based on the chloroplast genome sequences of *P. alaschanica* and other 14 species: *Castilleja paramensis* (KT959111), *Lindenbergia philippensis* (HG530133), *Orobanche crenata* (HG515537), *Pedicularis hallaisanensis* (MG770330), *Pedicularis ishidoyana* (KU170194), *Schwalbea americana* (HG738866), *Orobanche californica* (NC_025651), *Cistanche deserticola* (NC_021111), *Castilleja paramensis* (KT959111), *Rehmannia chingii* (KX426347), *Tectona grandis* (NC_020098), *Orobanche californica* (NC_025651), *Boulardia latisquama* (HG514460), *Conopholis Americana* (HG514459), *Epifagus virginiana* (EPFCPCG), *Phelipanche ramose* (HG803180).
